# Metabolic Reconstruction and Modeling of Nitrogen Fixation in Rhizobium etli

**DOI:** 10.1371/journal.pcbi.0030192

**Published:** 2007-10-05

**Authors:** Osbaldo Resendis-Antonio, Jennifer L Reed, Sergio Encarnación, Julio Collado-Vides, Bernhard Ø Palsson

**Affiliations:** 1 Bioengineering Department, University of California San Diego, La Jolla, California, United States of America; 2 Centro de Ciencias Genomicas, Universidad Nacional Autónoma de México, Cuernavaca, Morelos, México; The University of Tokyo, Japan

## Abstract

*Rhizobiaceas* are bacteria that fix nitrogen during symbiosis with plants. This symbiotic relationship is crucial for the nitrogen cycle, and understanding symbiotic mechanisms is a scientific challenge with direct applications in agronomy and plant development. *Rhizobium etli* is a bacteria which provides legumes with ammonia (among other chemical compounds), thereby stimulating plant growth. A genome-scale approach, integrating the biochemical information available for *R. etli*, constitutes an important step toward understanding the symbiotic relationship and its possible improvement. In this work we present a genome-scale metabolic reconstruction (iOR363) for *R. etli* CFN42, which includes 387 metabolic and transport reactions across 26 metabolic pathways. This model was used to analyze the physiological capabilities of R. etli during stages of nitrogen fixation. To study the physiological capacities in silico, an objective function was formulated to simulate symbiotic nitrogen fixation. Flux balance analysis (FBA) was performed, and the predicted active metabolic pathways agreed qualitatively with experimental observations. In addition, predictions for the effects of gene deletions during nitrogen fixation in *Rhizobia* in silico also agreed with reported experimental data. Overall, we present some evidence supporting that FBA of the reconstructed metabolic network for R. etli provides results that are in agreement with physiological observations. Thus, as for other organisms, the reconstructed genome-scale metabolic network provides an important framework which allows us to compare model predictions with experimental measurements and eventually generate hypotheses on ways to improve nitrogen fixation.

## Author Summary

Nitrogen fixation is an important process for improving plant development in crops. Overall, it constitutes a central role in the nitrogen cycle which is essential to life. In this work we were interested in understanding nitrogen fixation in Rhizobium etli from a genome-scale perspective. Using the genome annotation and scientific literature, we reconstructed the metabolic network for R. etli, a bacterium that fixes nitrogen. The reconstructed metabolic network was used to analyze how this network is utilized during nitrogen fixation. From this metabolic network, we built a model that was found to be in agreement with the general biochemical properties of *Rhizobia*, when it fixes nitrogen. Additionally, we have included an analysis of how gene deletions affect symbiotic nitrogen fixation. We propose that the metabolic reconstruction presented here can be useful as a theoretical template to understand and suggest a hypothesis for improving nitrogen fixation and its biochemical interaction with plants.

## Introduction

**Figure 1:**
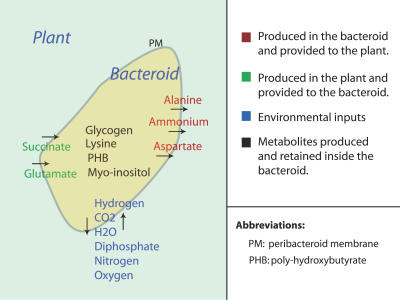
Nutrients Exchanged between Plant and Bacteroid Flow of molecules exchanged during symbiosis between plant and bacteroid. PM represents the peribacteroid membrane. The exchange of nutrients includes some amino acids whose evidence has been recently reported [6]. The presence of myo-inositol has been observed, but its source is unknown.

Free-living bacteria belonging to the *Rhizobium* genera are often symbiots associated with plants of the family *leguminosae*. These bacteria differentiate and have the ability to fix atmospheric nitrogen into ammonium when some compounds are exchanged between the bacteroid and its plant host [1–3]. Establishment of effective symbiotic nitrogen fixation between plant and bacteria is a complex process whose understanding constitutes an interesting scientific challenge with clear implications in plant development sciences and agriculture [4]. Nitrogen fixation in *Rhizobium* involves a complex plant–bacteria symbiotic relationship orchestrated by the genetic and metabolic networks of both organisms [3]. In general, the plant supplies carbon sources and glutamate to the bacteroid, while the bacteroid in turn provides the plant with ammonium, aspartate, and alanine [5,6] (see Figure 1). The exchange of these compounds creates a dependent symbiotic relationship between these two organisms whose effectiveness is essential to improving plant growth and bacterium survival.

*Rhizobium etli CFN42* is a nitrogen-fixing bacterium whose genome annotation has been reported recently [7]. Nitrogen fixation in R. etli occurs in the last of three developmental stages. The first developmental stage is related to the infection process of plant roots by *Rhizobium*. This begins when the plant excretes flavonoids which signal nodule formation [2]. The second stage is characterized by bacterial growth inside the plant, and the construction of a compartmentalized globular structure, called a nodule. This specialized structure protects nitrogenase, a key enzyme in nitrogen fixation, against irreversible oxidative damage by oxygen [5]. Finally, the last stage involves differentiation of the bacteria into a bacteroid able to reduce atmospheric nitrogen inside the nodule [8]. The computational analysis of this last stage is the main focus of this work.

Systemic understanding of nitrogen fixation in R. etli requires the construction of a model able to integrate genomic and high-throughput data in a hierarchical and coherent fashion [9]. Integrative models of this sort constitute a powerful and elegant strategy to study the mechanism of cell behavior. In particular, constraint-based models constitute such an approach, with a capacity to predict organism phenotypes operating at steady state [10–13]. Here, we present a reconstruction of the metabolic network in R. etli, the first reconstruction made for a *Rhizobium* organism. A constraint-based approach [12], including flux balance analysis (FBA) [14], is used to analyze the physiological capability of the bacterium when it fixes nitrogen. To show the utility of this analysis, the consistency between model predictions with experimental observations in some metabolic pathways is evaluated. Then we analyze the effects that some gene deletions have on symbiotic nitrogen fixation and compare them with available experimental observations. Experimental evidence on how these gene deletions affect nitrogen fixation activity are available for most cases investigated computationally, and it provides important information to validate our in silico modeling.

## Results

### Metabolic Network in Rhizobium etli

**Figure 2:**
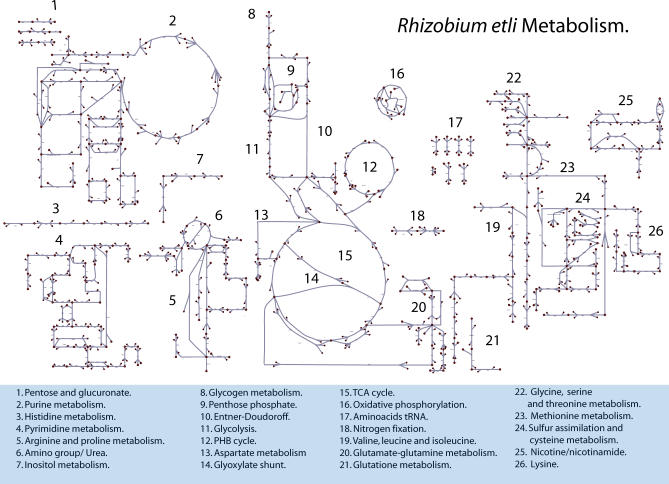
Metabolic Pathways for Rhizobium etli The metabolic reconstruction for *R. etli* includes 26 metabolic pathways involving 363 genes and 383 metabolic reactions.

The metabolic reconstruction was generated from the KEGG annotated genome sequence for R. etli [7], journal publications, automated reconstruction databases [15], and information found in biochemical textbooks on nitrogen fixation [1]. Thus, our metabolic reconstruction includes reactions with evidence from the genome annotation or with clear experimental evidence for *Rhizobia*. The resulting reconstructed metabolic network for R. etli includes 387 reactions involving 371 metabolites and 363 genes. This reconstruction spans 26 metabolic pathways involving central metabolism (44 reactions), amino acids metabolism (136 reactions), purine and pyrimidine metabolism (89 reactions), PHB synthesis (8 reactions), and nitrogen metabolism (19 reactions). The properties of the network and the complete set of metabolic reactions with their corresponding gene–protein reaction associations are available in Dataset S1, Table S1, and Dataset S2, respectively. Nomenclature used for metabolites is included in Dataset S3. Figure 2 shows a metabolic map of the pathways present in the reconstruction. The journal publications supporting our metabolic reconstruction are reported in Dataset S4. The gap analysis of the metabolic reconstruction is reported in Dataset S5.

### Defining an Objective Function for Symbiotic Nitrogen Fixation

The characterization of the physiological capability of R. etli and prediction of ways to improve nitrogen fixation in the bacteroid are central themes in this study. For our analysis, we assume that symbiotic nitrogen fixation between the plant and bacterium has reached the steady state. To simulate nitrogen fixation during symbiosis, we have constructed an objective function (OF) representing a set of chemical compounds whose production in the bacteroid is essential to make this symbiotic process efficient. Instead of incorporating biomass components (phospholipids, proteins, DNA, RNA) for the organism into the OF, as has been done for other organisms such as Escherichia coli, only compounds which are known or thought to be produced during symbiotic nitrogen fixation are included. The main reason for this is that the bacteroid does not grow during the stage where nitrogen fixation occurs, which is the life stage of interest in this study.

After reviewing the available literature, we can postulate an OF for use in FBA, which represents symbiotic nitrogen fixation in R. etli. This OF is based on the following physiological information.

1) Plant–bacteroid exchange of some amino acids during nitrogen fixation has been suggested to be a general mechanism in *Rhizobia*. Aspartate and alanine play a central role in the development of nodules and in efficient nitrogen fixation [6]. According to a recent hypothesis, aspartate and alanine are provided to the plant from the bacteroid, while glutamate is supplied by the plant to the bacteroid [6,16]. In this context, we postulate that symbiotic nitrogen fixation is closely related to the efficient supply of alanine and aspartate from the bacteroid to the plant. L-lysine has been reported in Bradyrhizobium japonicum bacteroids, a related organism to R. etli, and this amino acid was also included in the OF [17].

2) Nitrogen fixation is energetically costly requiring 16 ATP molecules to reduce a di-nitrogen molecule into two molecules of ammonium, which are then exported to the plant [2,5]. The transport of ammonium from the bacteroid to the plant is crucial for establishing symbiosis with the plant.

3) During nitrogen fixation, some *Rhizobia* accumulate poly-ß-hydroxybutyrate (PHB) and glycogen [18,19]. These compounds serve as storage for carbon that can be used when others are not present [20,21] . The presence of PHB has been verified in R. etli in the free-living and bacteroid states [22]. Even though the synthesis of PHB requires energy, PHB production is important because it reduces the levels of NAD(P)H which can repress the activity of some enzymes in the citric acid (TCA) cycle [21]. Nodules in R. etli are characterized as determinate nodules, and there is experimental evidence that glycogen and PHB molecules are produced during nitrogen fixation [5]. Thus, we consider the production of PHB and glycogen to be important components of the symbiotic nitrogen fixation OF.

The identified essential components produced by the bacteroid and exchanged with plant during nitrogen fixation are depicted in Figure 1. The OF used to represent symbiotic nitrogen fixation is a linear combination of all the components noted above.

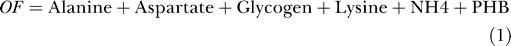



Thus, by maximizing this OF we suggest that during nitrogen fixation steady-state fluxes maximize the equal molar production of these compounds. In the rest of this work we will refer to the OF as the function that maximizes symbiotic nitrogen fixation. FBA consists of finding a flux distribution subject to steady-state mass balance and thermodynamic constraints, such that a linear OF is maximized [14]. FBA was performed (see Methods section), and the resulting flux distribution was compared with the known physiology of the organism. To verify the robustness of our results, we also ran the analysis using 1,000 different randomly weighted OFs (with weights selected from a uniform distribution) that did not assume equimolar contributions of the components shown in Equation 1 (see Methods section). We observed that the identified inactive metabolic pathways were independent of the stoichiometric coefficients of the OF (see Figure S1). The exchange reactions and the corresponding constraints used in simulations can be found in Dataset S2 and Table S2.

### Utilization of Central Metabolism

R. etli, like most other *Rhizobia*, fixes nitrogen in a microaerobic environment [23,24]. We simulated microaerobic conditions by constraining the upper bound of oxygen uptake rate to be 1 mmol/gDW/hr, a range estimated from experimental reports [25]. The simulations suggest that under microaerobic conditions, symbiotic nitrogen fixation activity is possible and some patterns for using the central metabolic pathways can be observed. During nitrogen fixation, dicarboxylates (succinate) provided by plant are metabolized via the TCA cycle. Efficient nitrogen fixation requires a high degree of coordination between the TCA cycle and oxidative phosphorylation [5].

**Figure 3:**
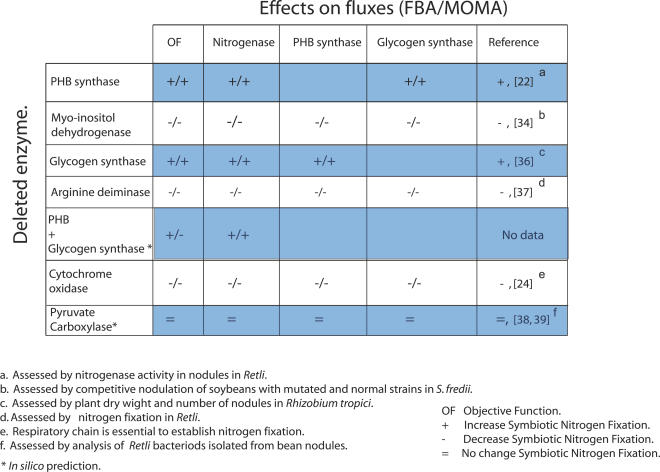
Experimental Validation with In Silico Gene Deletions The effects that mutations have on symbiotic nitrogen fixation was evaluated using linear (FBA) and quadratic (MOMA) programming [35]. This table shows the predicted increases (+) or decreases (−) in the OF, nitrogenase flux, PHB flux, and glycogen synthase flux with respect to the wild-type strain. Each entry of the table contains a pair of signs representing the effects of the deleted reaction on the fluxes. The first sign is the effect predicted by FBA, the second is that obtained from MOMA. At the end of each row we report the references that give experimental support to the increase or decrease of nitrogen fixation after the corresponding gene deletion. A brief description of the experimental assessment is presented in each gene deletion. In silico prediction of the effects of the double gene deletion (PHB and glycogen) depends on the optimization method (FBA or MOMA).

Bacterial respiration is important for nitrogen fixation for two reasons: it (1) reduces oxidative damage to nitrogenase by consuming oxygen, and (2) simultaneously generates ATP molecules needed for nitrogen fixation. R. etli is characterized by a branched respiratory chain which involves at least four terminal cytochrome oxidases [26,27] and whose activity is regulated by the oxygen concentration in the environment. Nonzero fluxes through oxidative phosphorylation were observed in the simulation results, indicating oxidative phosphorylation is needed to maximize symbiotic nitrogen fixation. In fact, removal of all cytochrome oxidase reactions results in a complete loss of symbiotic nitrogen fixation. This result is in agreement with the reported essentiality of respiration for nitrogen fixation [5,24] (see Figure 3).

The model predicts incomplete use of the TCA cycle. Experiments have reported the activity of all enzymes in the TCA cycle under microaerobic conditions in some *Rhizobium* bacteroids [5]; however, all TCA cycle enzymes are not always detected experimentally [20]. In addition, mutants in TCA cycle enzymes in B. japonicum are still able to fix nitrogen, suggesting that a complete set of TCA cycle enzymes is not strictly required to fix nitrogen [5,28]. Here, FBA supports the notion that incomplete use of the TCA cycle can still result in nitrogen fixation at low oxygen uptake rates in R. etli. The model predicts that citrate synthase, isocitrate dehydrogenase, and 2-oxoglutarate dehydrogenase are not used, which agrees with the enzymes suggested by experimental results to be inactive under microaerobic conditions [23]. This result is supported by flux variability analysis across alternate optimal solutions [29], and it was insensitive to changes in the weightings in the OF coefficients of metabolites in Equation 1. The flux through these enzymes was always zero in all optimal solutions, independent of the coefficients defined in the OF.

The model also predicts that there is no flux through the Entner-Doudoroff and pentose phosphate pathways under microaerobic conditions. As above, no flux through Entner-Doudoroff and pentose phosphate pathways was observed across all optimal solutions for 1,000 randomly weighted OF. Proteomic analysis of the B. japonicum bacteroid did not detect the presence of enzymes involved in the Entner-Doudoroff pathway in agreement with the model predictions [20]. However, the proteome data shows the presence of several of the pentose phosphate pathway enzymes, which contradicts the in silico predictions. One possible explanation for this discrepancy is that the use of this pathway could be strain-dependent. Thus, experimental measurements in *R. etli* bacteroids are needed to support or refute this computational result.

### Ammonium Assimilation Activity

It has been shown experimentally that the glutamine synthetase and glutamate synthase pathway (GS-GOGAT) constitutes the central mechanism of ammonium assimilation in free-living *Rhizobia* [30–32]. FBA predicts that there is no activity in the ammonium assimilation pathway during symbiosis (a non–free-living state), which agrees with recent reported measurements where ammonium assimilation was not observed during nitrogen fixation [20,32]. As before, flux variability analysis and random selection of the OF coefficients showed the inactivity of this metabolic pathway. The ammonium assimilatory pathways in the bacteroid are not active since most of the ammonium produced is transported to the plant. In fact, there is experimental evidence that an increase in ammonium assimilation negatively affects the nodulation process [32]. It also suggests that the inactivity of ammonium assimilation establishes optimal conditions for symbiotic nitrogen fixation, where complete transport of ammonium to the plant favors symbiotic nitrogen fixation.

### Myo-Inositol Utilization

Myo-inositol is an abundant compound in nodules and bacteroids, and it has an important influence on the efficiency of nitrogen fixation [33]. Although its origin is not well-understood, it constitutes an essential precursor for the synthesis of rhizopines. Rhizopines may function as osmotic protectants and in some *Rhizobium* strains confer a competitive advantage at early stages of nodulation [33,34]. The metabolic pathway of myo-inositol catabolism has been included in the metabolic reconstruction, and simulations with high uptake rates of myo-inositol suggest that it can be used as an energy source for nitrogen fixation. In silico analysis suggests that the activity of the myo-inositol dehydrogenase enzyme, encoded by *idhA*, increases symbiotic nitrogen fixation. Conversely, elimination of myo-inositol dehydrogenase enzyme from the network decreases the predicted nitrogen fixation activity. These model results have been observed experimentally in an *idhA* mutant in Sinorhizobium fredii [33]. The results obtained from simulations support the idea that myo-inositol catabolism plays an important role during nitrogen fixation, not only at early stages of nodulation but during nitrogen fixation as well. From a physiological point of view, there are some other possible explanations for reduced nitrogen fixation when the myo-inositol dehydrogenase enzyme is inactive. It may be a consequence of toxic levels of myo-inositol or a consequence of insufficient concentrations of myo-inositol for growth and maturation of the bacteroid [33]. Experimental data suggest that the essentiality of *idhA* is strain-dependent, and in this reconstruction we have included this metabolic pathway in agreement with the KEGG database [15]. From our in silico analysis, we observe that a deletion of myo-inositol dehydrogenase enzyme decreases nitrogen fixation activity, although it is not essential for symbiotic nitrogen fixation in R. etli. According to our in silico results, *idhA* is a key gene with potential implications for symbiotic nitrogen fixation.

### Gene Deletion Analysis

In this section, we evaluate the capacity of the model to predict the physiological behavior of the bacteroid when it suffers gene deletions. In silico analysis was conducted for deletions in PHB synthase, glycogen synthase, arginine deiminase, myo-inositol dehydrogenase, and pyruvate carboxylase. The first three enzymes are involved in PHB and glycogen synthesis and in arginine degradation, respectively. We have selected these enzymes because experimental results are available for *Rhizobium* on how these gene deletions affect symbiotic nitrogen fixation with respect to the wild-type strain.

To simulate gene deletions, the fluxes through reactions associated with the corresponding enzyme are constrained to zero, and the OF for FBA is constructed as before, excluding PHB or glycogen for the PHB and glycogen synthase deletion simulations, respectively (see Dataset S2). Gene deletion studies for E. coli have shown that the method of minimization of metabolic adjustment (MOMA) makes better mutant predictions than FBA [35]. Unlike FBA, MOMA identifies the flux distribution for the mutant strain that is closest to the wild-type flux distribution (measured as the Euclidean distance) [35]. Predicted changes in fluxes through some key reactions in the mutant strains versus the wild-type are reported in Figure 3, where mutant predictions were done using both FBA and MOMA. Qualitatively, FBA and MOMA made similar predictions for most mutants with respect to the wild-type symbiotic nitrogen fixation flux.

For the double gene deletion (PHB and glycogen synthase), FBA and MOMA predict different results. FBA predicts an increase in symbiotic nitrogen fixation, while MOMA predicts a decrease in symbiotic nitrogen fixation; unfortunately, no experimental results are available for the double deletion mutant. For this case, we observed slight variations in exchange fluxes when using the two analysis methods. There was also a set of biochemical reactions that have no flux in the FBA solution for the double mutant but are used in the MOMA solution: aconitase (ACONT), 2 oxoglutare dehydrogenase (AKGDH), citrate synthase (CS), cystathionine b-lyase (CYSTL), cystathionine beta-synthase (CYSTS), 2-dehydro-3-deoxy-phosphogluconate aldolase (EDA), 6-phosphogluconate dehydratase (EDD), and fructose bisphosphate aldolase (FBA). Conversely, we identified three reactions, methylmalonate (MMSAD3), inositol dehydrogenase (INS2D), and inositol catabolic reaction (INSCR), whose fluxes were active in FBA, but inactive in the MOMA solution. The complete flux distributions for the double mutant predictions are included in Dataset S6.

Simulations for deletions of PHB synthase predict that symbiotic nitrogen fixation increases, in agreement with the experimental observations in *R*. *etli* [22]. Experimentally, deleting PHB synthase increases NADH levels and consequently reduces the NAD+ / NADH ratio. A possible physiological explanation for the increase in nitrogen fixation rate could be that the increase of reductive power (lower NAD/NADH ratio) can be channeled to nitrogen fixation [22]. A similar effect on symbiotic nitrogen fixation is predicted for the glycogen synthase deletion, which also agrees with the observed physiological response reported for Rhizobium tropici [36]. FBA suggests that the same effect would occur for R. etli. The simulations predict that the flux through PHB synthase increases in the glycogen synthase mutant, and, similarly, the flux through glycogen synthase increases in the PHB synthase mutant. These results are in agreement with experimental reports suggesting that the quantities of both polymers are relatively flexible such that inhibition of one results in an accumulation of the other [22], a property that is qualitatively observed in our in silico modeling.

It is unclear whether arginine is supplied to the bacteroid by the plant, and as a result the metabolic network reconstruction lacks an arginine transport reaction and ornithine degradation reactions. Recent evidence has found that the arginine deiminase pathway is active in R. etli bacteroids which convert arginine into ornithine while generating ATP and CO_2_. By allowing the ornithine carbamoyl transferase reaction to be reversible and including an arginine–ornithine antiporter in the network, the symbiotic nitrogen fixation flux increased by more than 40% compared with the condition where arginine is not metabolized by the bacteroid (using *iOR363*). Deletion simulations removing arginine deiminase from this modified network predict a decrease in symbiotic nitrogen fixation (Figure 3), which is in agreement with experimental mutations of the *arcA* gene, which encodes the arginine deiminase enzyme in R. etli [37].

Pyruvate carboxylase is not used in the wild-type solution identified using FBA, so mutant predictions for pyruvate carboxylase are identical to the wild-type predictions. It is known experimentally for R. etli that deletion of this enzyme does not affect nitrogen fixation [38]; however, it does not discard the possibility that this enzyme may be important in other life stages or in rhizosphere competition [39,40].

To verify that these conclusions obtained from the gene deletion analysis are not sensitive to the coefficients in the OF, we randomly assigned OF coefficients for the five metabolites from a uniform distribution of values between 0 and 1. A total of 1,000 different random OF were subsequently used individually in the gene deletion analysis. The qualitative effect of the gene deletions on symbiotic nitrogen fixation was independent of the OFs used; for example, in all 1,000 cases the deletion of PHB synthase increased symbiotic nitrogen fixation. However, quantitative prediction of gene deletion effects was dependent on the chosen OF coefficients. To improve quantitative model predictions, a better approximation of the physiological levels of the OF components is needed.

### Phenotypic Phase Plane Analysis

Phenotypic phase plane (PhPP) analysis is a useful method to characterize the steady-state solution space projected in two dimensions [41,42]. Through this analysis, the steady-state flux distributions can be divided into a finite number of regions, each with similar metabolic flux patterns and characterized by equivalent shadow prices [41]. The shadow prices give us information on how the OF would change if the metabolites were additionally supplied to the network. These shadow prices can be used to classify the phenotypic phase plane into regions where the availability of different metabolites limits symbiotic nitrogen fixation.

**Figure 4:**
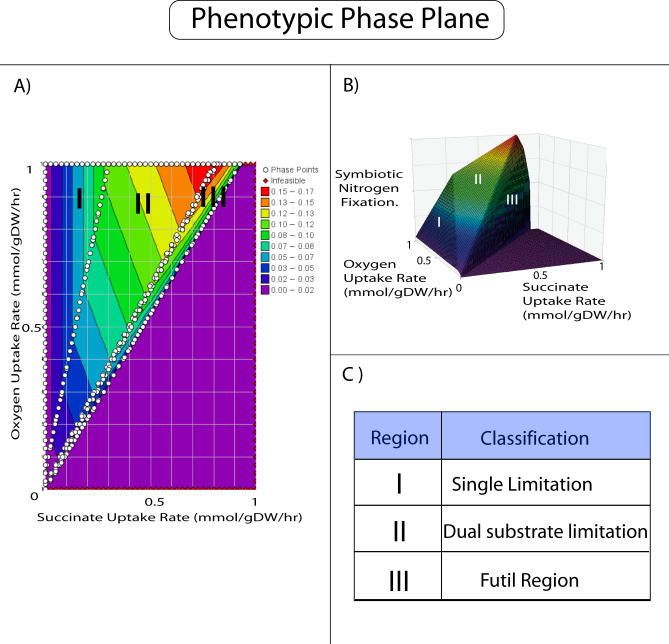
Phenotype Phase Plane The phenotypic phase plane shows the dependence of symbiotic nitrogen fixation on succinate and oxygen uptake rates. Succinate and oxygen uptake rates are expressed in mmol/gDW/hr. The OF has the same units, and its magnitude is expressed by the color scale (A). Regions denoted by I, II, and III characterize qualitatively different phenotypes in the phase plane (C). The phase plane is shown in 2-D (A) and 3-D (B).

The metabolic network for R. etli was analyzed with respect to two parameters, succinate and oxygen uptake rates. The low oxygen uptake rate inside the nodules is such that it creates a microaerobic environment optimal for nitrogen fixation [43,44]. Constraining our analysis to low uptake rates of oxygen (1 mmol/gDW/hr ), we found three regions in the PhPP, each one characterized by a qualitatively different optimal use of metabolic pathways [41] (see Figure 4). As shown in Figure 4, it is possible to identify a finite number of regions, each one characterized by the effect that oxygen and succinate uptake rates have on the OF. Region I is characterized by a single limiting substrate, succinate, which establishes an independent relationship between the OF and the oxygen uptake rate for a fixed succinate uptake rate. Region II has dual substrate limitations. In this region an increase in the uptake rate of either succinate or oxygen will increase symbiotic nitrogen fixation. Finally, in region III, an increase in succinate uptake rate produces a decrease in the symbiotic nitrogen fixation, while an increase in oxygen uptake rate increases the OF in region III. Region III is defined as a futile region [41]. The line of optimality represents the optimal relation between the substrate uptake rates and the OF. In this case, it lies on the boundary between regions I and II. Points on the line of optimality represent the optimal oxygen uptake required for the oxidation of succinate to maximize symbiotic nitrogen fixation [45].

In silico analysis shows that an increase in the succinate uptake rate, at a fixed oxygen uptake rate (1mmol/gDW/hr), increases symbiotic nitrogen fixation until a threshold in succinate uptake rate is reached (see Figure 4). At succinate uptake rates higher than this value, an inhibitory effect on symbiotic nitrogen fixation is observed. Here, the available oxygen is not enough to oxidize the excess succinate and it reduces symbiotic nitrogen fixation. This indicates that oxygen is a limiting compound in nitrogen fixation.

## Discussion

The contributions of this work are the metabolic reconstruction of R. etli (the first reconstruction made for *Rhizobia*) and the FBA of the resulting model during nitrogen fixation stages. Even with a lack of detailed experimental information, like kinetics constants and flux limitations for most reactions, we have been able to show that constraint-based methods can describe the capabilities of the metabolic network consistent with available experimental information. The construction of an OF that mimics symbiotic nitrogen fixation and qualitatively agrees with bacteroid physiology constitutes a substantial contribution in this work. Our OF was sufficient to obtain some of the main qualitative physiological characteristics for *R. etli* during nitrogen fixation. Thus, our model consistently reproduces, in agreement with the literature, the utilization of pathways like oxidative phosphorylation, gluconeogenesis, and PHB biosynthesis during nitrogen fixation. Additionally, the analyzed gene deletion set was in qualitative agreement with the response of the experimentally observed symbiotic nitrogen fixation activity (see Figure 3). These particular gene deletions were selected because their effects on nitrogen fixation activity have been measured experimentally, allowing us to evaluate our computational results. This reconstruction can be used to suggest gene deletions that could enhance symbiotic nitrogen fixation. Here we show one example where a double gene deletion in PHB synthase and glycogen synthase could potentially increase symbiotic nitrogen fixation.

The advantages of this in silico framework have been shown in other organisms [10,12,45], and we expect that for R. etli such computational analysis will be useful to design and improve nitrogen fixation in plant development and agriculture. This last point constitutes a valuable scientific objective which requires integration of experimental data to improve and update the metabolic reconstruction. For instance, aspartate aminotransferase in B. japonicus is essential, and its deletion is detrimental to nitrogen fixation [17], while a deletion of glutamine synthetase increases nitrogen fixation [17]. We do not observe this behavior in silico for *R. etli*. Experimental evaluation of these mutants in R. etli will provide a means to validate and improve the model.

Similarly, recent studies have shown the ability of *Rhizobia* to produce amino acids in well-defined environments and the effects that these amino acids have on symbiotic nitrogen fixation [16,17,46]. Although the production of the complete spectrum of amino acids has not been well-characterized in R. etli during nitrogen fixation, we have predicted their production with the model, and compared them with the amino acids experimentally observed in B. japonicum bacteroids [17]. FBA predicts the production of amino acids included in the OF (aspartate, alanine, and lysine). Experimental evidence shows the production of these amino acids in B. japonicum bacteroid [17]. Conversely, the experimental data for the B. japonicum bacteroid disagrees with the in silico analysis for R. etli, where asparagine, methionine, leucine, isoleucine, glycine, glutamine, and serine are not predicted by the model to be produced. Measurements of amino acid production in R. etli bacteroids are needed so a more accurate OF can be constructed [46]. Taken together, the reconstruction and analysis presented here provides an initial template for studying symbiotic nitrogen-fixing bacteria, and it can be used to generate hypotheses, design experiments, and to test predictable control principles for the metabolic network of *R. etli.*

## Methods

### Flux balance analysis.

Metabolomic flux prediction through the R. etli metabolic network was done using FBA [12]. We assume that all the chemical compound concentrations and fluxes are at steady state. To constrain the space of all the possible steady-state flux distributions, we impose stoichiometric mass balance constraints, thermodynamic constraints pertaining to reaction reversibility, and some enzyme capacity flux constraints [12]. Optimization of the OF was solved using the SimPheny software (Genomatica). MOMA calculations were carried out as described previously [35] using GAMS with only the metabolic and transport fluxes used in calculating the Euclidean distance; the exchange fluxes and OF values were omitted from the distance metric (see Datasets S1–S6). For the arginine deiminase mutant predictions, the following network modifications were made: ornithine carbamoyl transferase became reversible, an arginine–ornithine antiport reaction was included, and exchange fluxes for arginine and ornithine were added (a maximum arginine uptake rate of 5 mmol / gDW / hr was used).

### Exchange flux constraints.

The bacteroid is an open thermodynamic system that exchanges components with the plant through its peribacteroid membrane (see Figure 1). In our in silico analysis, we have classified chemical reactions into three categories: internal, exchange, and sink. The first contains most of the reactions in the metabolic reconstruction (all metabolic and transport reactions occurring inside the bacteroid). The second includes reactions which represent an interchange with the plant host (i.e., they allow metabolites to cross the system boundary). Finally, the third classification includes the entry or exit of metabolites by an unidentified source into the bacteroid. The only sink included is for myo-inositol, since the compound is observed in bacteroids, but it is unknown whether it is supplied by the plant or synthesized inside the bacteroid. In the case of the myo-inositol sink, we have limited the flux through the reaction so that myo-inositol present in the bacteroid can only be consumed. Most of the exchange reactions were defined as freely taken up or secreted, with the exception of arginine and oxygen. The oxygen uptake rate was limited by reported experimental measurements made in bacteroids [25]. The complete set of reactions and their flux constraints are available in Dataset S2 and Table S2.

### Reduced objective function for gene deletions.

In silico analysis of gene deletions for arginine deiminase, myo-inositol dehydrogenase, cytochrome oxidase, and pyruvate carboxylase was simulated by removal of the corresponding enzymatic reactions. In all these cases, the OF used was the same as the one presented in Equation 1. However, for glycogen synthase, PHB synthase, and the double mutant (PHB + glycogen synthase), new reduced OFs were used, where the corresponding component(s) (glycogen, PHB, or both) were omitted. For example, in the PHB synthase mutant the new reduced OF was:

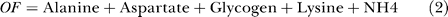
The optimization problems (FBA and MOMA) were then solved with these new reduced OFs. The OF for each gene deletion is presented in Dataset S2.

### Random assignment for the coefficients in the objective function and flux variability analysis.

To verify that the inactive metabolic pathways identified in this in silico analysis are not dependent on the stoichiometric coefficients defined in the OF, we performed flux variability analysis [29] combined with random assignment on the coefficient of the OF. We verified the robustness of our reported results for some enzymes in the following metabolic pathways: TCA cycle (aconitase A and B, isocitrate dehydrogenase, 2-oxoglutarate dehydrogenase, citrate synthase), Entner-Doudoroff Pathway (2-dehydro-3-deoxy-phosphogluconate aldolase, 6-phosphogluconate dehydratase), pentose phosphate pathway (glucose-6-phosphate dehydrogenase, 6-phosphogluconolactonase, phosphogluconate dehydrogenase, and ribulose 5-phosphate 3-epimerase), and ammonium assimilation (glutamate synthase, glutamine synthetase, and glutaminase). We assigned random numbers (from a uniform distribution between 0 and 1) for the stoichiometric coefficients defining the OF. After generating a randomly weighted OF, flux variability analysis was used to calculate the maximum and minimum flux values for each enzyme across all alternate optimal solution which maximizes this random OF. This procedure was repeated 1,000 times. This analysis was done using Matlab and LINDO.

## Supporting Information

Dataset S1 General Network Properties (13 KB XLS)

Dataset S2 Set of Reactions Included in Metabolic Reconstruction (100 KB XLS)

Dataset S3 Abbreviations of Metabolic Compounds (47 KB XLS)

Dataset S4 References Utilized for the Metabolic Reconstruction of Rhizobium etli
(82 KB XLS)

Dataset S5 Gap Analysis and Dead Compounds in the Metabolic Reconstruction (18 KB XLS)

Dataset S6 Flux Analysis Simulation Results for PHB + Glycogen Synthase double deletion. Only the fluxes which were nonzero in at least one calculated flux distribution are shown.
(23 KB XLS)

Figure S1 Statistic Distribution of Variability Analysis for Citrate Synthase This distribution was obtained from uniform random sampling (between 0 and 1) of the coefficients in the OF.
(197 KB PDF)

Table S1 Metabolic Pathways Included in the Reconstruction and Their Corresponding Number of Reactions Details are in Dataset S2.
(14 KB XLS)

Table S2 Exchanges Fluxes and Their Bounds Used in All FBA Simulations LB and UB denote the lower and upper boundary constraint.
(32 KB DOC)

## Abbreviations

FBA: flux balance analysis
mmol /gDW / hr: millimole per gram dry weight per hour
OF: objective function
PHB: poly β hydroxybutyrate
PhPP: phenotypic phase plane
TCA: tricarboxylic cyclic acid
