# Supplementary material for: Metabolic Reconstruction and Modeling of Nitrogen Fixation in Rhizobium etli
Source: PLoS Comput Biol. 2007 Oct 5;3(10):e192. doi: 10.1371/journal.pcbi.0030192 (PMC2000972; doi:10.1371/journal.pcbi.0030192)
Supplement: Supplementary file 1 [file pcbi.0030192.sg001.pdf]

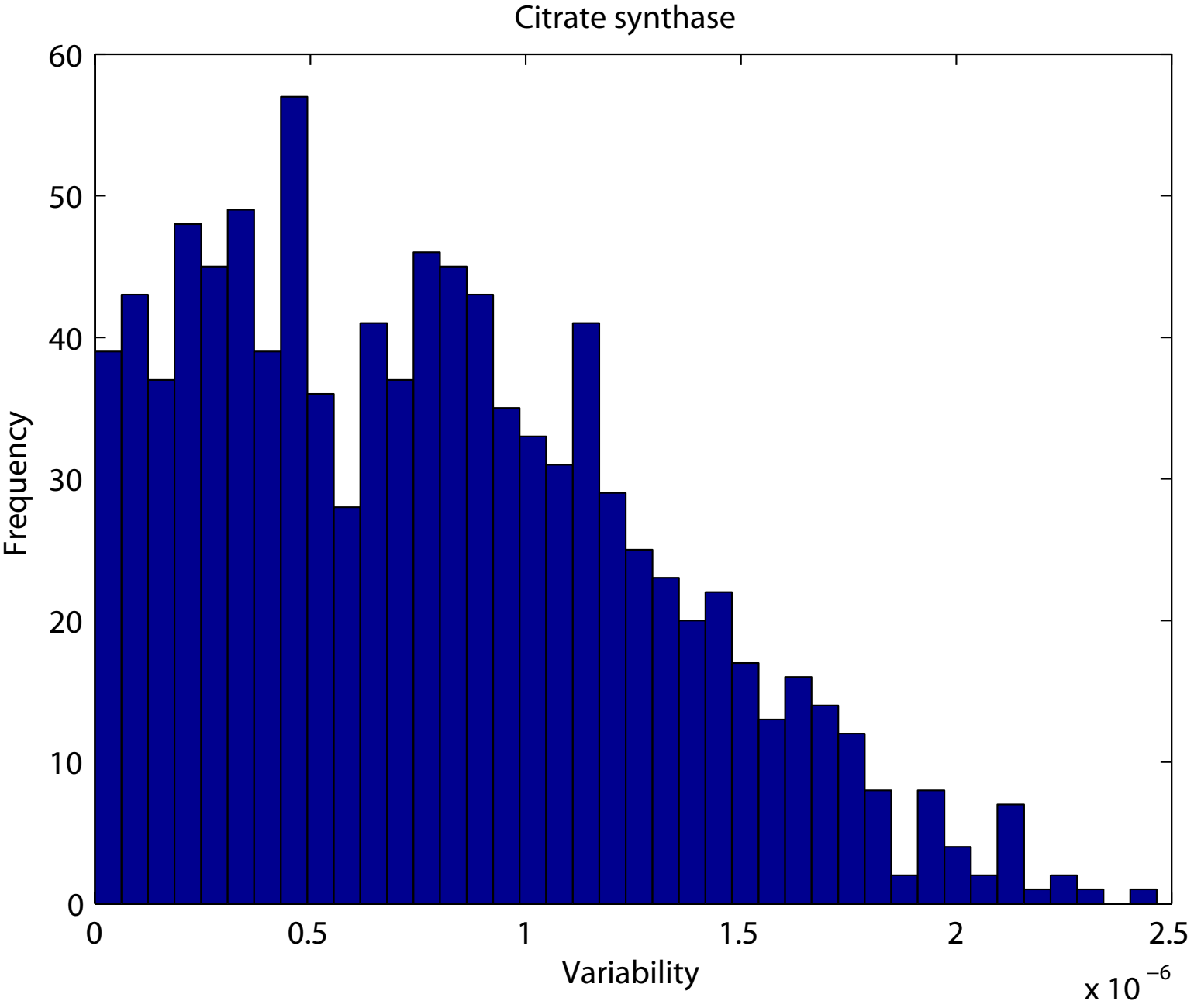

Citrate synthase
Frequency
Variability
x 10 −6
0
10
20
30
40
50
60
0
0.5
1
1.5
2
2.5
