# Supplementary material for: Metabolic Reconstruction and Modeling of Nitrogen Fixation in Rhizobium etli
Source: PLoS Comput Biol. 2007 Oct 5;3(10):e192. doi: 10.1371/journal.pcbi.0030192 (PMC2000972; doi:10.1371/journal.pcbi.0030192)
Supplement: Supplementary file 2 [file pcbi.0030192.st002.doc]

| **Exchange Flux** | **LB** | **UB** |
| --- | --- | --- |
| L-Alanine | 0 | ∞ |
| L-Aspartate | 0 | ∞ |
| L-Arginine | -5 | 0 |
| Co2 | -∞ | ∞ |
| Phosphate | -∞ | ∞ |
| Glutamate | -20 | ∞ |
| Hydrogen | -∞ | ∞ |
| Inositol | -0.01 | 0 |
| Nitrogen | -∞ | ∞ |
| Ornithine | -∞ | ∞ |
| Oxygen | -1 | 0 |
| Phosphate | -∞ | ∞ |
| Succinate | -∞ | 0 |
| H20 | -∞ | ∞ |
